# Advances in PET/CT Imaging for Breast Cancer

**DOI:** 10.3390/jcm12134537

**Published:** 2023-07-07

**Authors:** Dorine de Jong, Elise Desperito, Karine A. Al Feghali, Laurent Dercle, Romain-David Seban, Jeeban P. Das, Hong Ma, Abin Sajan, Brian Braumuller, Conor Prendergast, Connie Liou, Aileen Deng, Tina Roa, Randy Yeh, Antoine Girard, Mary M. Salvatore, Kathleen M. Capaccione

**Affiliations:** 1 Center for Cell Engineering, Memorial Sloan Kettering Cancer Center, New York, NY 10065, USA; ddejong990@gmail.com; 2 Department of Radiology, Columbia University Irving Medical Center, New York, NY 10032, USA; ed2202@cumc.columbia.edu (E.D.); ld2752@cumc.columbia.edu (L.D.); hym2103@cumc.columbia.edu (H.M.); abs9064@nyp.org (A.S.); braumuller.b@northeastern.edu (B.B.); cprender@student.nymc.edu (C.P.); col9043@nyp.org (C.L.); tir7004@nyp.org (T.R.); ms5680@cumc.columbia.edu (M.M.S.); 3 RefleXion Medical, Inc., Hayward, CA 94545, USA; kfeghali@reflexion.com; 4 Department of Nuclear Medicine and Endocrine Oncology, Institut Curie, 92210 Saint-Cloud, France; romaindavid.seban@curie.fr; 5 Laboratory of Translational Imaging in Oncology, Paris Sciences et Lettres (PSL) Research University, Institut Curie, 91401 Orsay, France; 6 Department of Radiology, Memorial Sloan Kettering Cancer Center, New York, NY 10065, USA; dasj@mskcc.org (J.P.D.); yehr@mskcc.org (R.Y.); 7 Department of Hematology and Oncology, Novant Health, 170 Medical Park Road, Mooresville, NC 28117, USA; adeng87@gmail.com; 8 Department of Nuclear Medicine, Centre Eugène Marquis, Université Rennes 1, 35000 Rennes, France; a.girard@rennes.unicancer.fr

**Keywords:** breast cancer, triple negative breast cancer (TNBC), ^18^F-FDG, PET radiotracers

## Abstract

One out of eight women will be affected by breast cancer during her lifetime. Imaging plays a key role in breast cancer detection and management, providing physicians with information about tumor location, heterogeneity, and dissemination. In this review, we describe the latest advances in PET/CT imaging of breast cancer, including novel applications of ^18^F-FDG PET/CT and the development and testing of new agents for primary and metastatic breast tumor imaging and therapy. Ultimately, these radiopharmaceuticals may guide personalized approaches to optimize treatment based on the patient’s specific tumor profile, and may become a new standard of care. In addition, they may enhance the assessment of treatment efficacy and lead to improved outcomes for patients with a breast cancer diagnosis.

## 1. Introduction

Breast cancer is now the most frequently diagnosed cancer in the world. Despite improvements in screening techniques and therapy, it is the leading cause of death from cancer among women aged 20 to 39 in the United States [1]. Breast cancer incidence is highly correlated with human development, and countries with the highest levels of human development have the highest incidences of breast cancer, although over half of breast cancer cases are diagnosed in low- and middle- income countries [2,3,4]. Age is the most important risk factor, and the incidence increases with age [5]. Other risk factors can be classified into reproductive and non-reproductive factors, both of which are influenced by economic development [6,7,8,9,10]. Breast cancer (BC) is a heterogeneous disease with different histological and molecular subtypes, including luminal A, luminal B, HER2-positive, and triple-negative (TN) breast cancer [11]. Together with different patient-related factors, the subtype influences disease management, response to therapy, and prognosis [11].

TNBC, which is characterized by the absence or low expression of estrogen receptor (ER) and progesterone receptor (PR) and a lack of HER2 overexpression, is the leading cause of breast malignancy death and accounts for approximately 15% of all breast cancers [12,13,14,15]. Until recently, treatment for TNBC was limited due to the lack of validated targeted therapies. However, newly approved drugs are ushering in a new era of treatment for TNBC [12]. In addition, various antigens found in TNBC are under investigation as potential small molecule, antibody, and CAR T cell therapy targets in light of the successes seen against other malignancies [12]. As treatments are evolving, the concomitant development of novel imaging techniques aims to provide physicians with better ways to visualize cancer and establish personalized therapeutic strategies.

## 2. Current Imaging Practices for Breast Cancer Screening, Monitoring, and Potential for Future Avenues

### 2.1. Imaging Modalities for Breast Cancer Screening

Imaging plays a central role in breast cancer for both screening and diagnosis. The American College of Radiology recommends annual breast cancer screening beginning at 40 years of age for average-risk women, i.e., patients with a lifetime risk of breast cancer <15%, with no established upper age limit [16,17]; 2D screening mammography is recommended, and whenever possible digital breast tomosynthesis (DBT), which is sometimes referred to as 3D mammography. In addition to planar images, DBT allows for the viewing of thin-section reconstructed images, yielding a higher cancer detection rate, particularly in women with dense breast parenchyma [18,19,20,21,22]. Adding ultrasound (US) to mammography increases cancer detection rate [23,24,25]. Indeed, dense breast parenchyma lowers the sensitivity of mammography and increases the risk of false negatives compared to patients with fatty breasts [26]. Supplementing mammography with molecular breast imaging (MBI) using ^99^Tc-Sestimibi SPECT and/or SPECT/CT imaging (commercially known as the Miraluma test) in this group of women increases the cancer detection rate [27,28]; however, whole-body radiation dose with this technique is limiting, particularly considering the need for repeated imaging studies [29]. Similarly, while positron emission tomography/computed tomography (PET/CT) with fluorine-18-2-fluoro-2-deoxy-D-glucose (^18^F-FDG-PET) can identify breast cancer, the associated high radiation dose limits its use in screening and diagnosis, and it is often reserved to assess the extent of disease. Breast MRI for average-risk patients lacks evidence supporting improved outcomes. For high-risk patients, MRI has demonstrated higher sensitivity than mammography, and the combination of mammography and MRI in this population has the highest sensitivity [30,31,32,33,34]. Of note, there is increased utilization of cross-sectional imaging with chest CT for evaluation of the lungs, with the additional opportunity for early detection of other diseases [35]. The breast parenchyma is imaged on chest CT, and should not be excluded from the scan reviewed by the radiologist [36]. High breast density can be reliably diagnosed on chest CT [37,38]. Retrospective research has demonstrated that three-dimensional chest CT is sensitive and specific for breast cancer diagnosis [39,40]. Future prospective studies are necessary to evaluate a possible role of chest CT in breast cancer diagnosis.

### 2.2. Imaging Modalities for Breast Cancer Monitoring

As mentioned above, imaging is an integral part of breast cancer early detection and evaluation of the extent of disease after it had been diagnosed. In women where there is a concern for advanced, metastatic, or recurrent disease, ^18^F-FDG PET/CT plays a key role in problem-solving with equivocal or suspicious findings and identifying nodal disease and/or distant metastases when used in addition to standard imaging. While demonstrating excellent sensitivity and specificity for certain subtypes of breast cancer, ^18^F-FDG PET/CT has inherent limitations.

**Figure 1:**
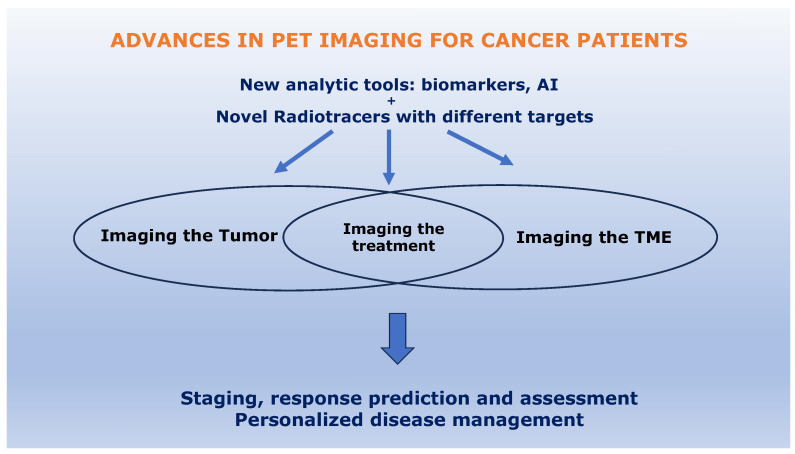
Strategies to improve PET/CT imaging of breast cancer patients.

^18^F-FDG transport into cells is mainly mediated by a Na^+^-dependent glucose transporter (GLUT). Increased uptake of ^18^F-FDG occurs in cancers due to overexpression of GLUT isotypes. However, it is not specific to cancer, and SUVmax varies among different subtypes of breast cancer, being the lowest for luminal subtypes and the highest for TNBC [41]. Furthermore, inflammatory cells and granulation tissues may demonstrate ^18^F-FDG uptake due to GLUT-1 and GLUT-3 overexpression from cytokine stimulation, which can lead to false positive signals, such as for mastitis [42,43,44]. A further limitation is that PET/CT lacks sensitivity in detecting small tumors <1 cm due to the inherent limitations of the imaging modality itself [45]. Awareness of these limitations is fueling the development of new imaging strategies to guide better care for different solid tumor types [46,47], including breast cancer patients (cf. Figure 1).

## 3. New Analytic Tools in PET/CT Breast Imaging

^18^F-FDG PET/CT plays an important role in staging breast cancer and subsequent restaging and monitoring response after treatment, with high sensitivity and specificity for disease beyond the breast [48,49,50]. To enhance the information available from PET imaging, the potential of AI and radiomic approaches to enhance the data provided by current ^18^F-FDG PET/CT imaging are being investigated, especially the role of metabolic parameters, as detailed below.

### 3.1. Tumor-Related Biomarkers

Several studies have demonstrated that biomarkers such as FDG uptake (SUVmax), metabolic tumor volume (MTV), and total lesion glycolysis (TLG) are associated with poor prognosis in the spectrum of histological subtypes of breast cancer [51,52,53] and at different stages of the disease [54,55,56]. Furthermore, changes in tumor SUVmax are strongly correlated with pathologic response in stage II or III breast cancer patients treated by neoadjuvant chemotherapy [57,58,59].

### 3.2. Non-Tumor-Related Biomarkers

Recent works suggest that measuring metabolic activity in non-tumor tissues could provide valuable biomarkers, such as measuring metabolism in lymphoid tissues (bone marrow and spleen) that are in close communication with the tumor microenvironment in order to predict response to therapy [60,61,62,63]. As the underlying pathophysiological mechanisms of breast cancer are currently being elucidated [64], studies imaging mouse models with breast cancer by ^18^F-FDG PET/CT could help to decipher pro-tumoral phenotypes and the pathways involved in therapeutic resistance in humans.

### 3.3. Novel Applications of FDG PET/CT-Tumor-Related Biomarkers

FDG uptake in the primary tumor may reflect the overall activity of the tumor microenvironment. Indeed, studies have shown that tumor SUVmax significantly and positively correlates with the degree of tumor infiltrating lymphocytes (TILs) and the expression of programmed cell death-ligand 1 (PD-L1), suggesting its potential use as a biomarker of immune checkpoint blockade therapies targeting PD-L1 [65]. Parameters extracted from ^18^F-FDG PET/CT have been associated with markers of tumor-related inflammation, including the neutrophil-to-lymphocyte ratio (NLR) and platelet-to-lymphocyte ratio (PLR), in large cohorts of patients with invasive ductal breast cancer [66,67]. Despite these promising studies, ^18^F-FDG PET/CT imaging is not routinely used to predict prognosis or the benefit that a patient could gain from adjuvant systemic therapies in early-stage invasive breast cancer. Several genomic signatures are available in this regard, including the widely used Oncotype DX, and provide a recurrence score (RS) with a probability of relapse at different time horizons [68]. Two studies have demonstrated that tumor SUVmax could be independently correlated to the RS [69,70]. New therapeutic molecules, antibody-drug conjugates (for example sacituzumab-govitecan) [71,72], and combined therapy regimens (for example, chemoimmunotherapy) [73,74] have revolutionized the management of breast cancer, particularly in patients whose prognosis is the worst among breast cancer types. Considering that there is not yet any effective predictive or prognostic metabolic biomarker for such novel therapies, it is of paramount importance to determine whether PET/CT imaging can contribute to the optimization of patient management

## 4. Novel Radiotracers for PET/CT Imaging of Breast Cancer

### 4.1. Promising Tracers for Tumor Imaging

While mainly striving for superior breast cancer detection and monitoring compared to ^18^F-FDG, these radiotracers may serve additional objectives, such as treatment selection. They may predict the efficacy of treatment response by imaging the target of interest before treatment. In addition, they can allow treatment monitoring of targeted therapies, and possess theragnostic functions. These PET agents have promising capabilities in imaging of many cancer types, such as PD-L1, while others are more breast specific (FES); see Table 1.

#### 4.1.1. PD-L1

PD-L1 is commonly expressed on the surface of antigen-presenting cells and tumor cells. The expression of programmed cell death protein 1 (PD-L1) has been demonstrated to be associated with a pathway that drives lymphocyte exhaustion. It is used by cancer cells to escape immune surveillance. Several immune checkpoint inhibitors that target either PD-1 or PD-L1 are on the market, with others in development [75].

This provides a strong rationale for showing whether PD-L1 PET could be used to predict responders and monitor response to therapy. The current reference standard for the evaluation of PD-L1 expression is immunohistochemistry. Its main advantage is that it can quantify PD-L1 expression on both tumor cells and immune cells. However, it is an invasive technique with sampling bias due to tumor heterogeneity. The limited sample obtained via biopsy can not evaluate the intratumor temporal and intertumor spatial heterogeneity of PD-L1 expression [76]. Radiolabeled PD-L1 PET imaging can non-invasively quantify tumor pharmacokinetics, biodistribution, and intratumor/intertumor temporal/spatial heterogeneity. A limitation of this approach is that, due to the limited spatial resolution of PET, it cannot discriminate PD-L1 expression on tumor cells versus immune cells. Radiotracers targeting the PD-1/PD-L1 axis are being rapidly developed [77], such as the high-affinity engineered protein scaffold (HAC-PD-1) [78], anti-PD-L1 antibodies [79,80,81], anti-PD-1 [79,82], and small non-antibody PD-L1-specific peptides [83]. Radionuclides used in the radiolabeling of these agents targeting PD-1 or its ligand include either the positron emitters ^64^Cu [79] and ^89^Zr [81] or the single photon emitters ^111^In [80] and ^99^mTc [83]. The advantage of non-antibody small molecules with high affinity for PD-L1 [79] over anti-PD-L1 antibodies is that they have higher tumor penetration, higher signal-to-noise ratios, faster image acquisition within a few hours after injection, lower injected activities, and faster clearance by the kidneys [79,83].

#### 4.1.2. EGFR and Her 2

The human epidermal growth factor receptor (EGFR) family (also known as receptor tyrosine kinase (RTK) Class I, ErbB family, or HER-family) is a class of transmembrane tyrosine kinase receptors. This family is composed of four receptors (HER1-4) which are expressed in a variety of tissues of epithelial, mesenchymal, and neuronal origin, where they are involved in fundamental cellular processes such as proliferation, differentiation, migration, invasion, and angiogenesis [84,85]. EGFR receptors are linked to human cancer pathogenesis. HER1 (also known as EGFR) and HER2 are mutated in many cancers, and have been extensively studied because of their oncogenic potential and the potential to exploit them as therapeutic targets [84,85]. Overexpression of the EGFR family of receptors has been demonstrated in breast cancer, and is associated with a worse prognosis [86,87]. Selectively targeting this family of receptors for therapy requires methods of identifying patients with HER expression who could benefit from these therapies and monitoring them over time for changes in expression that may correlate with resistance to therapy. A potential benefit of whole-body imaging of EGFR receptor expression is the ability to detect intra- and interlesional heterogeneity [88].

Clinical trials investigating EGFR-targeted therapies for breast cancer, including monoclonal antibodies (panitumumab and cetuximab) and small molecule inhibitors (gefitinib, erlotinib, and afatinib), have not been very promising [89,90,91]. One possible explanation is that EGFR may be translocated from the cell surface to the nucleus, thereby reducing drug/receptor interaction and contributing to drug resistance [92,93]. Transport of EGFR to the nucleus is reportedly mediated through Src hyperactivation [94,95]. Studies have shown that blockade of Src kinase activity halts EGFR translocation to the nucleus and can increase its availability in the plasma membrane, thereby improving cetuximab sensitivity in TNBC [96]. This explains the importance of monitoring changes in EGFR distribution within the cellular milieu (nucleus versus plasma membrane) post-Src targeted inhibition. This monitoring can be performed using EGFR immune-PET. Recently, zirconium-89 (^89^Zr)-labeled antibodies were investigated for use in imaging EGFR expression in vivo [97,98,99]. ^89^Zr-cetuximab, for example, has shown promise in visualizing tumors expressing EGFR in murine models. The uptake of ^89^Zr-cetuximab in dasatinib-treated versus control untreated TNBC tumor-bearing mice has been compared, showing that dasatinib-treated cells display higher binding of ^89^Zr-cetuximab [100]. ^89^Zr-cetuximab uptake increased by at least 1.4-fold following dasatinib treatment. The authors concluded that cetuximab PET could be used to monitor and quantify EGFR expression and cellular distribution as well as to measure the effects of inhibition of Src kinase on EGFR, thereby potentially guiding treatment decisions in patients with TNBC [100]. c-MET is another tyrosine kinase receptor closely linked to EGFR that is overexpressed in TNBC. Amivantamab is a human bispecific antibody that has been engineered to target both EGFR and c-MET simultaneously, and is currently in phase I trials for metastatic non-small cell lung cancer (NCT02609776) [101]. ^89^Zr-labeled amivantamab has been successfully investigated as a PET-imaging CDx in preclinical models of TNBC to assess the combined expression of EGFR and c-MET as well as the delivery of amivantamab to TNBC tumors for therapy [102]. Future clinical studies will help to establish whether this agent could be used to select patients for the treatment of TNBC.

Her2 is overexpressed in 10–25% of patients with breast cancer, and is linked to poor prognosis [103,104,105,106,107,108]. The development of HER2-targeted treatments using humanized anti-Her2 receptor monoclonal antibodies, trastuzumab, and pertuzumab has significantly improved survival of patients with Her2-positive breast cancer subtype [109,110]. Despite the success of Her2-targeted therapy, it remains a challenge due to innate or acquired resistance and because of spatial and temporal heterogeneity in Her2 expression [111,112,113]. About 10% of patients have inconsistent Her2 status between primary and metastatic sites [113]. Thus, there is a need for accurate characterization of Her2 expression, as currently the two methods used to determine Her2 status in breast cancer (immunohistochemistry and fluorescence in situ hybridization) fall short of depicting this heterogeneity [114]. Molecular imaging with anti-Her2 probes enables non-invasive whole-body assessment of Her2 expression and permits selection of patients for whom HER2-targeted treatments, along with therapeutic response monitoring and assessment of changes in Her2 expression over time, will be of benefit [115,116]. Agents that have been evaluated for Her2 imaging are mostly based on the HER-2 targeting antibody trastuzumab. A first-in-humans ^89^Zr-trastuzumab PET imaging clinical study in patients with metastatic breast cancer allowed determination of the optimal dosage and timing of administration of the monoclonal antibody ^89^Zr-trastuzumab [117]. In this trial, ^89^Zr-trastuzumab showed excellent tumor uptake and visualization of Her2-positive metastatic lesions 4–5 days after injection. Trastuzumab-naïve patients required a 50 mg dose of ^89^Zr-trastuzumab for optimal biodistribution, and patients already on trastuzumab treatment required a 10 mg dose. Uptake of ^89^Zr-trastuzumab has been observed in metastatic brain lesions as well, though usually monoclonal antibodies such as trastuzumab cannot cross an intact blood–brain barrier because of their large size, limiting their therapeutic efficacy against brain metastases [117]. No adverse events related to ^89^Zr-trastuzumab injection were observed, and the total estimated radiation dose was 18 mSv, comparable to two abdominal CT scans or one ^18^F-FDG PET scan [117,118]. The Zephir study showed that pretreatment imaging of Her2 with ^89^Zr-trastuzumab is a promising tool for studying interlesional heterogeneity in advanced breast cancer. When combined with early ^18^F-FDG PET/CT after one cycle of trastuzumab emtansine (T-DM1), it can predict which patients will benefit from this therapy, with a positive predictive value and a negative predictive value of 100% [119]. A study showed that ^89^Zr-trastuzumab PET imaging influenced diagnosis and clinical decision-making for patients with Her2-overexpressed breast cancer; ^89^Zr-trastuzumab PET altered treatment decisions in 40% of patients and increased physicians’ confidence in the existing treatment plan in 50% of cases. In addition, it improved physicians’ understanding of the disease in almost 90% of cases [120]. Initial clinical results with the smaller Her2-directed F(ab′)2 fragment ^68^Ga-DOTA-F(ab′)2-trastuzumab were promising as well, demonstrating its feasibility and safety [121]. Other studies have demonstrated the feasibility and efficacy of ^64^Cu-DOTA-trastuzumab in identifying Her2-positive lesions in patients with primary and metastatic breast cancer as well as in predicting response and outcomes of patients with metastatic breast cancer receiving T-DM1 [122,123]. Using short-lived positron emitters such as ^68^Ga and ^64^Cu may ultimately allow sequential noninvasive quantitation of Her2 expression using PET.

#### 4.1.3. HDAC

Among the most frequent epigenetic changes are histone modifications [124]. In this context, histone deacetylase (HDAC) enzymes play a critical role in cancer by deacetylating histone and non-histone proteins, which are involved in the regulation of the cell cycle, DNA damage response, metastasis, and other cellular processes [125]. In humans, eighteen HDAC enzymes have been discovered. Studies have shown overexpression of HDAC1 in gastric and breast cancer [126]. High levels of HDACs are associated with advanced disease and poor outcomes [125]. In light of the prevalent role of HDACs in tumorigenesis, HDAC inhibitors (HDACi) such as vorinostat and panobinostat have been developed and are currently FDA-approved to treat hematologic malignancies. In breast cancer, HDAC enzymes play an important role in the regulation of estrogen- and progesterone-mediated signaling. In small clinical trials, it has been shown that HDACi alone does not alter survival outcomes in patients with breast cancer; however, when used in conjunction with hormone inhibitors there is improvement in progression-free survival and overall survival outcomes [126]. The underlying mechanism is that the HDACi resensitizes the cancer cells to hormonal treatment, as 50% of hormone-positive breast cancers develop resistance to hormonal therapy throughout treatment [126]. With the promise of using HDAC inhibitors as a new adjuvant therapy for breast cancer, there is interest in determining which patients would benefit from the additional therapy. Mapping the overexpression and distribution of HDACs using a radiolabeled HDACi (^11^C-Martinostat) and PET/CT has found extensive application in brain imaging [127,128]. Future studies are looking to quantify the amount of HDACs in breast tumors to guide therapy and assess response to treatment.

#### 4.1.4. FES

The estrogen receptor-specific PET radiotracer ^18^F-fluoroestradiol (^18^F-FES) works by selectively binding to the estrogen receptor alpha (ER-α). Approximately 80% of all breast cancers are ER^+^ [129]. FES is more specific in diagnosing ER^+^ cancers than traditional ^18^F-FDG due to its lack of background activity. Several recent studies have demonstrated the advantages of ^18^F-FES in the diagnosis of breast cancer over more standard imaging with ^18^F-FDG. A study held at Fudan University Shanghai Cancer Center imaged nineteen patients with both FDG and FES, evaluating 245 lesions observed with one or both radiotracers. They found that 41 lesions (16.7%) were exclusively detected by ^18^F-FES PET scan yet absent in ^18^F-FDG PET scanning [130].

FES PET is especially effective in diagnosing ER^+^ invasive lobular carcinoma (ILC). The most common histology of breast cancer is invasive ductal carcinoma (IDC), which comprises 80% of diagnosed breast cancer cases, while the second most common histology is ILC, which comprises 10–15% of cases. ILC is known to be more difficult to diagnose than IDC due to its low cellular density per unit volume and poor ^18^F-FDG PET avidity. Detection of ILC with mammography, ultrasound, MRI, and ^18^F-FDG PET/CT can be very challenging. Because ILC is nearly always (95%) ER^+^, FES PET has proven to be very sensitive for its detection, and as such has the potential to improve PET detection and staging of this type of breast cancer.

In addition, FES PET has proven to be more sensitive in the diagnosis of metastatic lesions than ^18^F-FDG PET (90.8% vs. 82.8%, respectively) [130]. This study suggests that FES could be used in concert with FDG for a more comprehensive view of metastasis and their progression [128]. In a group of seven patients exhibiting ER^+^ ILC, 268 osseous lesions were found between FES and FDG. Of the 268 lesions, 253 (94%) were ^18^F-FES avid and 90 of 268 (34%) were ^18^F-FDG avid [131]. FES has proven to be more sensitive in diagnosing malignancies than FDG. FES-PET can be beneficial in determining whether patients will respond to estrogen receptor-targeted therapy as well.

Although more research is needed to confirm the specificity of FES imaging, early studies have shown that it is a highly promising agent for patients with ER+ ILC.

#### 4.1.5. Mucin 1

Mucin 1, or MUC1, is a human epithelial surface glycoprotein encoded by the gene *MUC1*, and has emerged in recent years as a promising tumor-specific antigen in breast cancer [132]. Although MUC1 is overexpressed in a variety of cancers, such as lung, prostate, and gastrointestinal tract neoplasms, it is of particular interest in breast cancer, as it is expressed in over 90% of all breast cancers and about 94% of triple negative breast cancers [133]. MUC1 expression has been associated with tumor aggressiveness, and measurement of serum MUC1 levels by CA15-3 assay is a reliable predictor for breast cancer prognosis [134].

MUC1 is a type 1 transmembrane heterodimer with an α-subunit consisting of an extracellular N-terminal domain, a β-subunit composed of a transmembrane helix, and a short cytoplasmic tail [135]. The extracellular domain of MUC1 is defined by the presence of the amino acid sequence PDTRP, which is the same sequence recognized by several highly tumor-specific anti-mucin monoclonal antibodies [134]. Studies have successfully utilized the PDTRP sequence to synthesize novel MUC1-derived peptides and coupled it to a Gly-Gly-Cys (GGC, triamide-thiol) chelating sequence to facilitate radiolabeling in PET imaging [136].

While traditional markers such as ER, PR, and HER2 remain the mainstay for breast cancer workup, multiple studies have reported on the success of ^99^Tc-labeled MUC1 peptide for the targeted imaging of MUC1-positive breast cancer [137,138]. GGSK-1/30 is a monoclonal antibody which has been synthesized to specifically identified the MUC1 glycopeptide pattern on MUC1-positive human breast cancer cells [139]. It has highly specific immunohistochemical staining for malignant cells in TNBC and HR-positive cells as well. Radiolabeling of GGSK-1/30 with ^89^Zr in preclinical mouse models for breast cancer demonstrated tumor-specific accumulation resulting in high-contrast PET imaging [139]. A radiolabeled MUC1-conjugated folate hybrid peptide has been developed as well, serving as a dual-receptor-targeting imaging probe for breast carcinoma imaging [140].

It has been reported that 10% of breast cancers and as many as 70–80% of metastatic TNBCs express folate receptor α. Compared to the radiofluorinated MUC1 monomeric peptide, the unique radiofluorinated MUC1-conjugated folic acid (FA) hybrid peptide demonstrated superior affinity to breast cancer cells and better pharmacokinetics [138]. These observations were confirmed by PET imaging, and highlight the potential for combining MUC1 with other receptors to maximize tumor uptake.

In addition to its PET imaging capabilities, targeting MUC1 has been studied for breast cancer treatment. There are multiple ongoing clinical trials evaluating MUC1 peptide-based cancer vaccines [141,142,143]. The low immunogenic response and favorable biokinetics of MUC1-positive cells have led to the growth of multiple MUC-based monoclonal antibodies. Phase I and II trials have evaluated AS1402, a humanized IgG1 mAb binding MUC1, which induces antibody-dependent cellular cytotoxicity against MUC1-positive breast cancer cells [144,145]. Additional studies have targeted trastuzumab-resistant breast cancer cells with humanized MUC1 antibody (HzMUC1), which has been shown to inhibit the growth of HER2-positive breast cancer cells in animal trials [146].

#### 4.1.6. Tissue Factor

Tissue factor (TF) is a common surface target receptor in several types of solid cancers, including breast cancer, and is recognized as a potential therapeutic target in TNBC [12,147].

TF is a transmembrane protein that binds coagulation factor VII (FVII) with high affinity. While TF is selectively expressed in normal human breast tissue, it is overexpressed in select breast cancer tissues [148,149]. FVII is an enzyme primarily synthesized and secreted by hepatocytes that is involved in the extrinsic coagulation cascade [150,151]. The active form of this complex (TF-FVII) is upregulated in many solid tumors, leading to thrombin generation and hemostasis and resulting in cancer cell signaling, inhibition of apoptosis, promotion of cell migration, tumor angiogenesis, and metastatic spread through thrombin generation and PAR1 signaling [150,151,152,153]. TF is highly expressed in several human TNBC cell lines, such as MDA-MB-231, MDA-MB-468, and HCC-1806 [154]. High levels of TF have been found to contribute to disease progression in TNBC patients [149,150,151,155], and TF levels can act as a prognostic marker, as high TF expression usually contributes to a decreased overall survival rate in TNBC patients [153].

Prior studies have shown that targeting TF is a valid strategy for several malignancies other than breast cancer [156,157,158,159]. Both in vitro TNBC cancer lines and in vivo tumor xenografts in mice have shown that TF was overexpressed on cells and tumor neovasculature in up to 85% of TNBC patients and TNBC cell line-derived mice xenografts, while it was not detected in adjacent normal breast tissue. A second-generation TF-targeting immunoconjugate, L-ICON1, was able to kill TNBC cells in vitro via antibody-dependent cell-mediated cytotoxicity, which could make TF a useful target for the development of immunotherapies for TNBC patients [160].

Factor VII, the natural ligand to TF, can be radiolabeled to allow for specific imaging of TF, and as such could be used in imaging and therapy radionuclides for TNBC [153,154]. As for other tumor markers, assessing tumor TF status based on tissue sampling may be limited by both the invasive nature of biopsy and by tumor heterogeneity, especially in the context of metastatic disease [153,154]. Several pre-clinical studies have explored the utility of radiolabeling TF to evaluate TNBC. The first TF PET imaging was performed on subcutaneous xenograft mouse models using a ^64^Cu-labelled anti-TF antibody, ^64^Cu-NOTA-ALT-836. The authors found that in the highly TF-expressing model, imaging with ^64^Cu-NOTA-ALT-836 led to high tumor uptake at up to 48 h post-injection. ^64^Cu-NOTA-ALT-836-Fab exhibited high targeting efficiency in the MDA-MB-231 TNBC model [161]. Subsequently, another study utilized PET imaging in a TF-positive TNBC xenograft mouse model to examine the specificity of ALT-836, a TF targeting chimeric anti-human monoclonal antibody (mAb) fragment that binds to the factor X-binding site in TF. It demonstrated rapid and persistent tumor uptake on serial PET imaging. The TF specificity of the mAb tracer was validated both by histology and by reverse transcription-polymerase chain reaction test [154]. These results highlight the potential benefit of mAb Fab fragment imaging, including rapid tumor uptake and rapid blood clearance primarily through the hepatobiliary system, allowing superior tumor contrast in malignancies with abundant blood flow such as TNBC, which could facilitate same-day immunoPET imaging in clinical settings to improve TNBC patient management.

While antibody-based imaging agents possess optimal tumor accumulation, a significant obstacle for intact antibody PET imaging is prolonged circulation half-life [154,162,163]. A novel PET radiotracer for imaging of TF using ^18^F to label factor VII has been described, although in a non-TNBC setting [164]. The authors used FVIIai (which binds to TF with an affinity approximately five times greater than FVIIa [165]), which was radiolabeled with ^18^F by N-succinimidyl 4-^18^F-fluorobenzoate (^18^F-ASIS) and purified. Notably, the uptake of ^18^F-ASIS measured in vivo by PET imaging correlated with TF protein level measured ex vivo, while the uptake of ^18^F-ASIS correlated with TF expression measured ex vivo in tumor homogenates [164]. The authors concluded that, with new therapeutic agents targeting TF being developed, ^18^F-ASIS warrants further consideration as a TF-specific PET imaging agent with potential as a companion diagnostic for emerging TF-targeted therapies [164]. In addition, ^18^F-FVIIai presents fast pharmacokinetics, demonstrating its promise as a PET radiotracer for specific and noninvasive imaging of tumor TF expression [164].

A first-in-humans trial using ^18^F-ASIS PET imaging has been conducted on ten cancer patients, including three patients with breast malignancy. The mean ^18^F-ASIS plasma half-life was 3.2 h, with the radiotracer predominantly cleared by the renal collecting system [166]. Overall, ^18^F-ASIS appeared to be safe, with no adverse events observed in the patient cohort. This trial represents an important first step towards the introduction of ^18^F-ASIS PET imaging in the clinical evaluation of TF expression [166].

#### 4.1.7. CD146

CD146, known as melanoma cell adhesion molecule (MCAM), is a transmembrane glycoprotein which is expressed in breast cancer [167]. It is known as an epithelial–mesenchymal transition inducer, and plays an important role in triple negative breast cancer in high tumor stages with poor prognosis [168,169]. In triple negative breast cancer patients, CD146 is expressed at high levels in tumor cells and is associated with resistance to endocrine therapy. CD146 is hypothesized to promote breast cancer progression by induction of EMTs via the activation of RhoA and upregulation of Slug [168].

YY146 is a CD146-specific monoclonal antibody. Labeled YY146 is a highly selective molecule for imaging in triple negative breast cancer patients [168]. YY146 can be labelled with one of three radiotracers: ^52^Mn, ^89^Zr, or ^64^Cu. ^52^Mn-DOTA-YY146 presented a rapid and high (95%) yield labeling after 60 min incubation [168]. Liver and bone marrow had the highest off-target uptake. When labeled with ^89^Zr (with a half-life of 78.4h), YY146 biodistribution was similar. The biodistribution of ^89^Zr-Df-YY146 in tumor animal models was similar to ^52^Mn-DOTA-YY146 at all time points except in the liver at 4 h [170]. Animal studies using ^52^Mn-DOTA-YY146 demonstrated tumor uptake levels on PET imaging, and CD146 expression levels were consistent among multiple breast cancer cell lines. Studies with ^64^Cu-labeled YY146 have evaluated the tumor expression of CD146 in breast cancer cell line xenograft models using MCF-7 cells, ZR-75-30, SKBR3, MDA-MB-435, and MDA-MB-231 cells in BALB/c nude mice [171]. ^64^Cu-NOTA-YY146 PET imaging demonstrated that radiotracer uptake is correlated with protein levels in vitro. In lung metastasis orthotopic mice models, the MDA-MB-435 lung metastatic tumor demonstrated significantly higher radiotracer uptake than other breast cancer models [171].

**Table 1:** Examples of promising tumoral PET radiotracers under investigation.

Targets	Radiopharmaceuticals	References
PD-L1		
	[64Cu]-NOTA-PD-L1	[79]
	[89Zr]-atezolizumab	[82]
EGFR		
	[89Zr]-cetuximab	[100]
	[89Zr]-trastuzumab	[117]
	[64Cu]-DOTA-trastuzumab	[122]
HDAC		
	[11C]-Martinostat	[127]
Estrogen receptor		
	[18F]-FES	[129]
Tissue factor		
	[64Cu]-NOTA-ALT-836-Fab	[161]
	[18F]-ASIS	[166]
Mucin 1		
	MUC1-FA-[18F] SFB	[140]
CD146		
	[52Mn]-DOTA-YY146	[170]
	[89Zr]-Df-YY146	[170]
	[64Cu]-YY146	[170]
Trop 2		
	[89Zr]-DFO-AF650	[172]
Nectin 4		
	[68Ga]-N188	[173]

### 4.2. Additional Tumoral Radiotracers Being Investigated

Many tracers are currently being investigated. Among them, Trophoblast 2 (TROP 2) and Nectin-4 are other examples of exciting targets for breast cancer that need further investigation. Trop-2 is found at high levels in multiple cancers, including TNBC. ^89^Zr-labeled anti-Trop-2 antibody (AF650) has demonstrated strong preclinical results in pancreatic cancer detection [172]. Nectin-4 is a connective tissue adhesion molecule which is highly expressed in breast and bladder cancers. Human trials using a Nectin-4 radiolabeled tracer in bladder cancer have shown its potential as diagnostic tool for treatments targeting nectin-4 [173]. As a ligand of TIGIT, Nectin-4 presents the advantage of being a tumor-specific antigen that can be combined with immune checkpoint inhibition.

On the other hand, preliminary preclinical PET agents targeting CXCR4 and CMLK, which are highly expressed in breast cancer, have not demonstrated significant improvement at this stage.

Chemokine receptor 4 (CXCR4) is a G protein-coupled chemokine receptor that is expressed at significantly higher levels by breast cancer cells. Higher CXCR4 expression has been found to be associated with more extensive breast cancer lymphatic metastases and subsequently reduced survival [174]. Chemokine receptor CXCR4 has been explored as a target for PET imaging as well, although the results were less promising than for CD146 targeted agents [175]. ^68^Ga-pentixafor is a PET imaging agent designed to target CXCR4. It was tested on thirteen patients with breast cancer with modest uptake and visualization of tumors, demonstrating its feasibility; however, the results were not significant [172]. Future studies must confirm whether CXCR4 PET imaging can play a role in breast cancer diagnosis and monitoring.

Chemokine-like receptor 1 (CMLKR1) is a G-protein coupled receptor which binds to the ligand chemerin. CMLKR1 is known to be a player in inflammation via chemerin-mediated recruitment of macrophages and dendritic cells. Although its involvement in cancer cell migration is not well studied, its increased expression has been observed in invasive breast cancers and is associated with longer relapse-free survival; thus, CMLKR1-targeting markers may be a potential target for imaging and therapy [176]. A study tested potential chemerin-based CMLKR1 tracers in mouse models using novel DOTA-conjugated peptides labeled with radioactive ^68^Ga or ^177^Lu. Xenografts from the human breast cancer cell line DU4475 were used due to their high expression of CMLKR1, demonstrating that the tracer had significant affinity for CMLKR1, enabling PET detection; however, it had unfavorable kinetics [177].

### 4.3. Promising Tracers Targeting the Tumoral Microenvironement (TME)

PET imaging agents able to characterize the tumor microenvironment and/or allow real-time visualization of treatments are highly desirable. Many are currently in development, as they could provide a better understanding of resistance mechanisms and ultimately lead to early prediction of treatment response (see Table 2). Novel treatments may result in new radiological features that could be used as biomarkers of response.

#### 4.3.1. FAPI

Fibroblast Activation Protein (FAP) is a serine protease expressed in activated fibroblasts, which are found in cancer and other disease states yet not in normal tissue [178,179]. In the context of cancer, FAP expression is restricted to fibroblasts, not being expressed in cancer cells themselves [179]. Considering that FAP-expressing fibroblasts are found ubiquitously in cancer, targeting FAP for PET imaging and molecular targeted radiotherapy is a rational strategy. Indeed, the success of targeting FAP has been shown across multiple cancer types, including cancers that have been traditionally difficult to image with ^18^FDG-PET [180]. Several FAP-targeting molecules with a quinolone backbone and different R group substitutions have been tested to optimize binding characteristics. Two lead compounds, FAPI-02 and FAPI-04, have demonstrated excellent FAP targeting properties [181].

Continued development of these promising compounds has led to multiple preclinical studies, providing data to support the further translation of these compounds. One such study evaluated the potential of ^68^Ga-FAPI-04 PET imaging in patient-derived pancreatic cancer xenografts, finding that ^68^FAPI-04 displayed superior tumor-to-background uptake than ^18^FDG-PET [182]. Another study evaluated the theranostic combination of ^64^Cu-FAPI-04 and ^225^Ac-FAPI-04, demonstrating excellent tumor targeting, although in most organs ^64^Cu-FAPI-04 accumulation was significantly higher than ^68^Ga-FAPI-04, suggesting that the latter may be superior for FAP PET imaging. Administration of ^225^Ac-FAPI-04 significantly decreased tumor growth in a PANC-1 xenograft mouse model, serving as key support for the translation of this theranostic combination for pancreatic cancer [183]. In addition to FAPI-02 and FAPI-04, other FAP-targeting molecules have been developed, such as FAP-2286 [184] and UAMC1110 [185,186], in the search for compounds with the most optimal binding characteristics and tumor retention. Similarly, compounds in the quinolone FAPI series described above continue to be developed and refined for FAP PET imaging and targeted radiotherapy; such work has resulted in the development of FAPI-46, the leading ^68^Ga-labeled compound, and FAPI-74, the leading ^18^FDG-labeled compound, which are currently in clinical trials currently. Recently, a study evaluated the utility of FAPI-74 PET/MRI imaging for breast cancer to assess the ability of FAP PET to identify breast lesions seen on the accompanying MRI. The results demonstrated strong FAPI-74 accumulation in the breast cancer of all eighteen patients, with clear delineation of the tumor across different histologic types, receptor expression, and grades, supporting its use. Further, the same study showed lymph node involvement in preoperatively identified lymph node metastases, and in certain cases FAP PET uptake sites affected management decisions [187]. Together, these data support the use of FAP PET imaging in breast cancer, and suggest that it may play a key role in diagnosing and staging of cases that are not well-imaged using ^18^F-FDG PET. Both FAPI-46 and FAPI-74 are under commercial testing, with large-scale studies gathering data on side effect profiles and target specificity, all of which have demonstrated favorable characteristics. The excellent tumor-to-background ratio and ability to image tumors traditionally not well seen on ^18^FDG PET has led to much speculation as to whether FAP PET imaging might ultimately replace ^18^FDG PET [188,189,190]. In parallel with FAP PET imaging agents, therapeutic agents have been developed, as described above in the case of pancreatic cancer. A recent preclinical study comparing the efficacy of ^177^Lu-FAPI-46 against ^225^Ac-FAPI-46 found similar anti-tumor efficacy; although the effects of ^177^Lu-FAPI-46 were slower, they lasted longer [191]. Other research has investigated the role of FAP-targeting radionuclides against melanoma and lung cancer, finding similar excellent anti-tumor efficacy [192]. Clinical research has demonstrated that ^90^Y-FAPI-46 is a viable alternative radiotherapeutic in a case series in nine patients with advanced solid tumors (osteosarcoma, sarcoma, pancreatic cancer), finding little clinically significant toxicity and with approximately 50% of patients achieving disease control [193]. Considering that FAP is expressed across many tumor types, clinical trials aimed at broad tumor types are currently underway; these may represent a new possibility for patients with tumor types that lack effective therapeutic options, including triple negative breast cancer. While only time can tell the extent to which FAP-targeting theranostics will become part of the clinical algorithm for breast and other cancers, promising early data suggest that they could play in important role in disease diagnosis and control.

#### 4.3.2. Imaging Immune Cells

Visualization of the tumor microenvironment using PET could add prognostic value thanks to the ability to perform whole-body imaging, overcoming the limitation of sample bias due to the tumor heterogeneity which occurs with biopsies. For example, evaluating the density of different immune cell subsets via biopsy allows for the calculation of an immunoscore (IS) and can guide clinicians toward more aggressive approaches in case of low IS. CD3 and CD8 cell densities both in invasive margins (IM) and at the center of the tumor (CT) are used to provide a prognostic score from 0–4 [194]. The evaluation of the immunoscore through biopsy in a study of 103 breast cancer patients revealed a significant prognostic and potentially predictive role, particularly in TNBC [195].

PET imaging may serve as a tool for determining a more precise IS. Indeed, CD3+ and CD8+ T cell PET imaging agents have already been developed. Examples include ^89^Zr-DFO-CD3, which aims to predict immune response to therapy with CTLA-4 checkpoint inhibitors [196] and ^89^ZED88082A [197] or ^18^F-GEH200521, which have been developed to image CD8^+^ T cells dynamics in the context of immune checkpoint inhibitor treatment (NCT05629689). There is a need for new imaging tools to guide immunotherapy clinical trials for oncology patients [198].

In addition, type 2 tumor-associated macrophages (TAMs) could be a target of interest for PET imaging. TAMs are known to be involved in tumor progression, in particular due to their immunosuppressive role, and high TAM infiltration is correlated with poor patient prognosis [199]. TAM-targeted therapies (e.g., CSF-1R inhibitors, arginase inhibitors, PD-1 inhibitors) have been developed to modulate the immune system within the TME. Hence, TAMs could be used in PET imaging for tumor profile characterization, to guide the choice of immunotherapy, and to allow real-time treatment monitoring [200]. To date, several PET tracers targeting macrophages initially developed for inflammatory diseases have been tested in vivo [201]. Studying the polarization, depletion, and recruitment of TAMs using PET imaging could provide a better understanding of their role in cancer.

#### 4.3.3. Imaging the Treatment in Real-Time: Monitoring CAR T Cell Therapy

In light of the clinical success of Chimeric Antigen Receptor (CAR) T cell therapy for hematological malignancies, preclinical research and clinical trials are booming for solid tumors, including breast cancer [202]. The majority of investigated antigen targets for breast cancer CAR T cell therapy are tyrosine kinase receptors (e.g., HER2, EGFR, cMET, ROR1) and cell surface proteins (e.g., MUC1, mesothelin, ICAM1, folate receptor α) [202]. However, monitoring CAR T cells by quantification in the peripheral blood after infusion does not show their biodistribution or activation status in the tissues.

PET is an ideal whole-body imaging modality that allows both the functional status and the spatiotemporal dynamics of immune cells to be monitored. For example, ^64^Cu-labeled synTac (synapse for T-cell activation) can distinguish antigen-specific CD8^+^ T cells from bystander CD8^+^ T-cells [203]. Granzyme B PET imaging could serve as a quantitatively predictive (secreted) biomarker of response to cancer immunotherapy, [204] and the use of Zirconium-89-deferoxamine-ICOS monoclonal antibody as a PET tracer has demonstrated in vivo visualization of donor T cell activation in target tissues [205]. Various approaches have been investigated to monitor T cell dynamics with high specificity and sensitivity, including direct labeling of cells in vitro, proteins and peptides targeting endogenous T cell surface and secreted biomarkers, small-molecule metabolic tracers, and engineering cells to express PET reporter genes [206,207].

Considering the rise in CAR T cell therapy utilization in hematological malignancies and the intensive research to extend the treatment to various types of solid tumors, there is keen interest in developing PET imaging agents to monitor these cells in vivo [208]. Although radiolabeling appears relatively straightforward for CAR T-cell therapy, where the cells are expanded ex vivo, the incorporation of radionuclides can cause toxicities such as radiolysis and adversely impact T cell function. The radiolabel itself becomes diluted as cells divide and proliferate in vivo, reducing the utility of this approach for longitudinal imaging with real-time follow up of response [209,210]. PET imaging can provide a powerful means of measuring biological changes such as metabolism, cell location, and tumor burden. T cell tracking systems that combine T cell-specific probes with highly sensitive PET imaging allow longitudinal PET imaging and quantification of T cell dynamics.

In breast cancer, monitoring native T cells may play an important role in predicting response to therapy. To date, several PET radiotracers based on T cell metabolism have been developed for imaging the immune system, and even specific cell types such as activated T cells. One concern when designing T cell-specific PET probes is the overlap in the metabolism of T cells located in lymph nodes and intratumorally, which could result in on-target off-tumor imaging. Enzyme-based PET reporter genes have been widely explored to track T cells. T cell-specific PET imaging can visualize tumor-infiltrating lymphocytes and monitor the dynamics of T cells in response to chemotherapy, radiotherapy, molecular targeted therapy, immunotherapy, and adoptive cell transfer. ImmunoPET tracers using nanobodies or antibody fragments and short-lived PET radionuclides may enhance the target-to-background ratio and reduce the radiation dose [207].

#### 4.3.4. Imaging Hypoxia and Vasculature

Hypoxia is a common characteristic of solid tumors and a trigger for angiogenesis. There are different degrees of hypoxia and it varies across tumor types. Mapping hypoxia using PET imaging could have multiple applications, including identifying individuals with poor prognosis (as it increases resistance to radiotherapy and systemic therapy and alters antitumor immunity) who could benefit from therapies fighting tumor hypoxia. Indeed, several studies have shown that high uptake of hypoxia PET tracers such as [18F]-FMISO, [18F]-FAZA, [18F]-FETNIM, and [18F]-HX4 can predict poor treatment response and prognosis [211]. Longitudinal imaging of hypoxia has revealed that it can be considered an early predictive biomarker of therapeutic response. Hence, these agents are starting to be investigated as companion tools to guide radiotherapy and immunotherapy [212,213]. While hypoxia or matrix metalloproteinases are indirect markers of angiogenesis, VEGF, integrins, and fibronectin represent various targets for angiogenesis PET imaging [214].

**Table 2:** Examples of PET radiotracers under investigation to image the tumoral microenvironment.

Target	Radiopharmaceuticals	Reference
Fibroblast		
FAP	[68Ga]-FAPI-04	[182]
	[225Ac]-FAPI-46	[191]
	[177Lu]-FAPI-46	[191]
T cell		
CD3	^89^Zr-DFO-CD3	[196]
CD8	^9^ZED88082A	[197]
	[18F]-GEH200521	[198]
Activated T cell		
Synapse for T cell activation	[64Cu]-synTac	[203]
T cell activation in tissue	[89Zr]-deferoxamine-ICOS	[205]
Granzyme B	[68Ga]-NOTA-GZP	[204]
TAM		
Folate receptor	[18F]-AzaFol	[201]
Arginase	[18F]-FMARS	
CSF-1R	[11C]-AZ683	
Hypoxia		
pO_2_ < 10 mmHg	[18F]-HX4	[211]
	[18F]-FMISO	
	[18F]-FAZA	
Angiogenesis		
Integrin	[18F]-F-Galacto-RGD	[214]
VEGF	[89Zr]-Bevacizumab	
NGR tripeptide	[64Cu]-labelled NGR	

## 5. Conclusions

Due to the high incidence and mortality of breast cancer, continued development of imaging and therapeutic agents is needed to gain control of this disease. Imaging plays a central role in guiding clinicians in the diagnosis and management of the disease. ^18^F-FDG PET/CT remains the pivotal standard of care tool used in staging, restaging, and monitoring treatment response. Although more information is now being extracted from ^18^F-FDG PET/CT imaging with the advent of radiomics, there remains much room for improvement.

Here, we shed light on promising tracers beyond FDG that are currently in preclinical or clinical development. In addition to providing accurate tumor localization, tracers for estrogen receptors, EGFR, HDAC, PD-L1, or Tissue Factor could help clinicians to accurately determine treatment suitability and efficacy of targeted therapy. In addition, Mucin1, CD146, and immune cell markers may be part of the future personalized medicine armamentarium offered to breast cancer patients. Finally, thanks to its excellent target-to-background ratio and sensitivity, FAPI-specific PET agents could play a key role as versatile oncological radiotracers, complementing or perhaps replacing FDG.

The literature on the role of PET/CT imaging to guide precision medicine approaches in breast cancer is a dynamic landscape, with advances holding immense potential for enhancing diagnostic accuracy, individualizing treatment approaches, and improving patient outcomes in breast cancer management, although many of these advances need prospective validation in large clinical trials.

## Data Availability

Not applicable.
